# Appraisal of Clinical Improvement With Total Knee Arthroplasty in Valgus Knee Deformities: A Case Series

**DOI:** 10.7759/cureus.58039

**Published:** 2024-04-11

**Authors:** Alok KV, Garipalli Ramesh, Arjun S Chakrapani, Sanyam Akheja

**Affiliations:** 1 Trauma and Orthopaedics, Osmania Medical College, Hyderabad, IND; 2 Orthopaedics, Apollo Speciality Hospitals, Perungudi, Chennai, IND

**Keywords:** range of motion, medial parapatellar approach, functional outcomes, radiological assessments, osteoarthritis, valgus knee deformity, total knee arthroplasty

## Abstract

Introduction

This case series explores the efficacy of unassisted total knee arthroplasty (TKA) in addressing valgus knee deformity secondary to osteoarthritis. The study aims to evaluate functional outcomes pre- and post-surgery using the Knee Society Score (KSS) and radiological assessments in short-term follow-up. Six patients underwent TKA and were evaluated retrospectively. Statistical analysis revealed significant improvements in the angle of deformity, KSS, and range of motion postoperatively. The study underscores the success of TKA in correcting valgus deformity, improving knee function, and enhancing patient satisfaction.

TKA is a highly successful treatment for osteoarthritis, providing functional recovery and improved quality of life. However, valgus knee deformity presents a challenge in TKA, affecting approximately 10% of patients. This study aims to assess the functional outcomes of TKA for valgus deformity using KSS and radiological evaluation in short-term follow-up.

Materials and methods

A retrospective observational study was conducted from November 2022 to December 2023, involving six patients with valgus knee deformity secondary to osteoarthritis. TKA was performed with no technological assistance, and patients were assessed pre- and post-surgery using KSS and radiological measurements. Statistical analysis was performed using paired t-tests.

Case description

Six patients with grade two valgus deformity underwent technology-unassisted TKA. The postoperative assessment revealed significant improvements in the tibiofemoral angle, KSS, and range of motion. The medial parapatellar approach for TKA was utilized with a standard unconstrained primary TKA prosthesis, resulting in successful correction of deformity and improved knee alignment.

Discussion

TKA is a widely performed and reliable surgical intervention, with valgus knee deformity posing specific challenges. This study demonstrates the effectiveness of conventional TKA in correcting valgus deformity, improving knee function, and enhancing patient satisfaction in a very small case series. Comparison with previous studies supports the findings of the pre-existing literature, highlighting the importance of appropriate surgical approach and patient selection.

Conclusion

TKA utilizing a medial parapatellar approach proved effective in our small case series in correcting valgus deformity, improving knee function, and enhancing patient satisfaction. Short-term follow-up reveals significant improvements in stability, posture, and KSS scores. Further assessments and longer-term follow-up are warranted to confirm the long-term effectiveness of this approach.

## Introduction

One of the most successful areas of medical expertise is orthopaedic surgery, with rapid and innovative advancements in both surgery and treatment. Total knee arthroplasty (TKA) is a very successful treatment for osteoarthritis, producing positive outcomes in terms of functional recovery overall, good survival rates, and the ability to resume daily activities [1].

Valgus knee deformity is a challenge in TKA [2]. Almost 10% of patients treated with TKA were patients with valgus deformity, defined as a valgus angle greater than or equal to 10% [3]. The deformity can be congenital or result from osteoarthritis, rheumatic conditions, post-traumatic arthritis, and excess correction following a valgus osteotomy [4].

The characteristics of the valgus knee include patellar malalignment, tibial metaphyseal valgus remodelling, lateral tibial plateau bone loss, hypoplastic lateral condyle, and other conditions such as femoral head deformity [5]. According to the descriptions provided by Ranawat et al. and Lombardi et al. [3,6], the valgus deformity is typically divided into three grades (Ranawat classification) with a deviation of up to 10° indicating grade 1, which comprises 80% of valgus knees and which can often be rectified with lateral soft tissue releases. This repair technique is only useful in cases where the medial collateral ligament is intact. Grade 2 valgus deformity is characterized by functioning but contracted lateral soft tissue and an axis deviation between 10° and 20°. Grade 3 patients have a deviation axis of more than 20° and significantly compromised stabilizing medial knee tissues, necessitating restricted prostheses [7].

The amount of constraint required for stability correlates with the valgus deviation degree. For grade 1-2 valgus, the posterior stabilized (PS) prosthesis may be utilized; for grade 3 patients, a fully constrained TKA is typically required [6]. Compared to a well-aligned knee, valgus knee deformity complicates regular TKA and increases the failure rate [8]. Therefore, the present study aims to evaluate and compare the functional outcomes before and post-TKA surgery using a medial parapatellar approach and a less constrained prosthesis for valgus deformity in patients with osteoarthritis using the Knee Society Score (KSS) [9,10] and radiological and clinical evaluation in a short-term (one year) follow-up.

## Materials and methods

A retrospective observational study was done from November 2022 to December 2023, involving six clinically and radiologically confirmed cases of valgus knee deformity who were scheduled for TKA at the Department of Orthopaedics, Osmania General Hospital. Patients who satisfied the following eligibility requirements were invited to take part in the study: (1) primary knee osteoarthritis with no history of prior knee surgery; (2) no uncontrolled systemic diseases; (3) no neurological, cardiac, psychiatric, or other medical conditions that would seriously impair physical functions (e.g., stroke); and (4) adequate language skills for communication, memory retention, and the ability to follow verbal instructions. Any of the following led to the exclusion of patients: (1) unwilling to participate; (2) if they were undergoing TKA for a condition other than osteoarthritis, like rheumatoid arthritis (RA). Six patients who were aware of the study purpose and met the inclusion criteria with the Institutional Ethics Committee's approval were followed for further analysis.

Methodology

**Figure 1:**
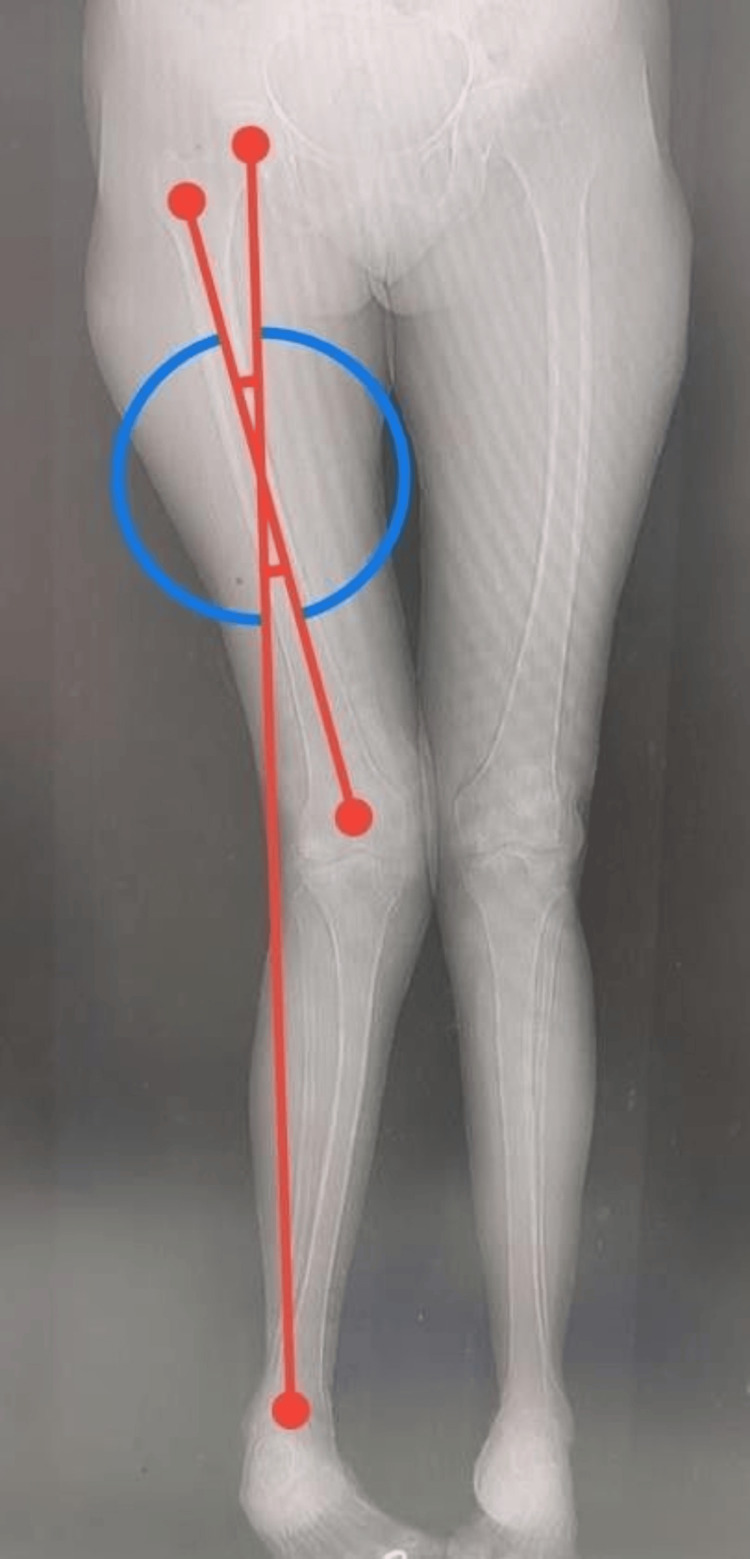
Preoperative scanogram angle measurement

**Figure 2:**
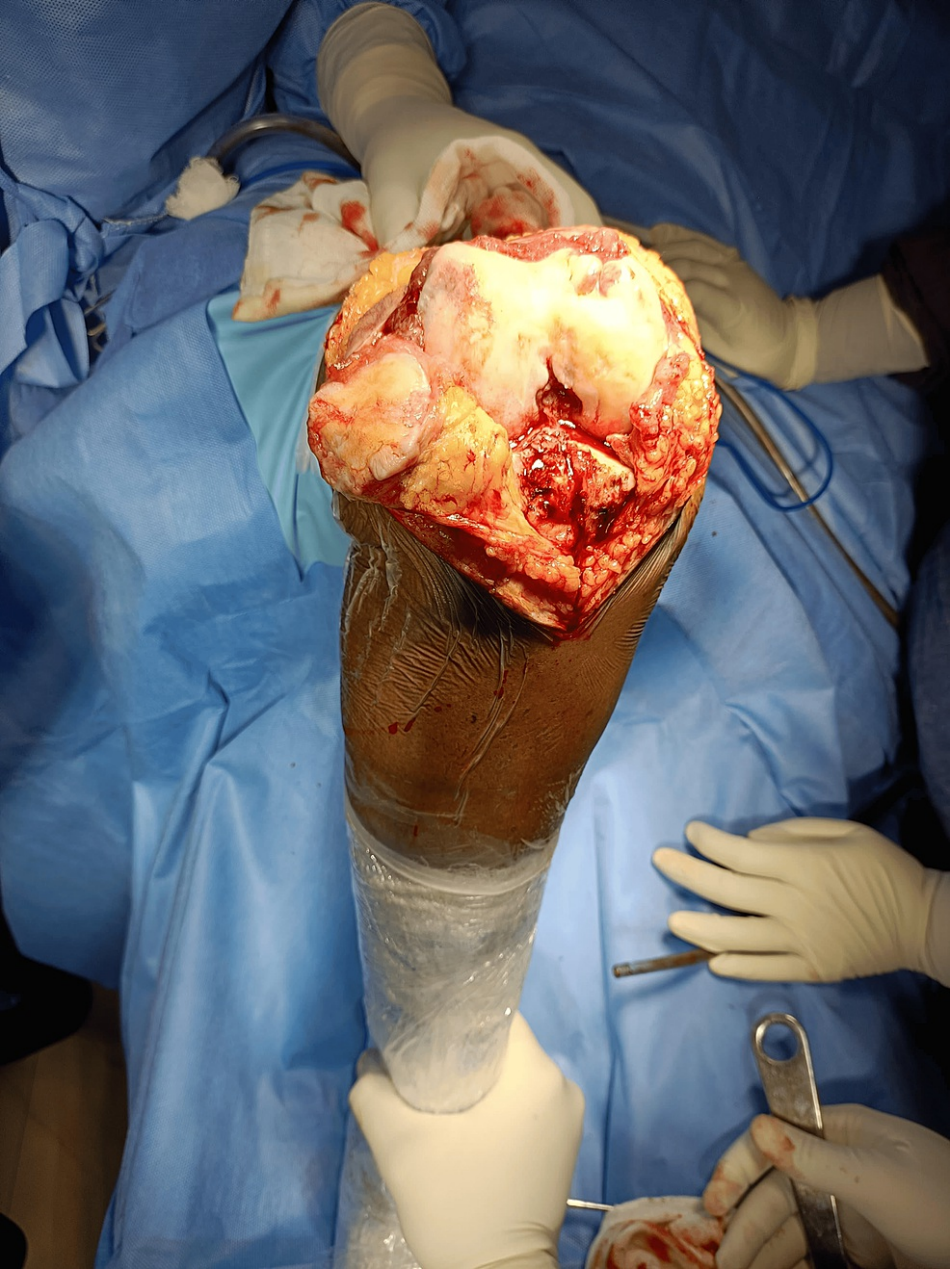
Medial parapatellar approach

**Figure 3:**
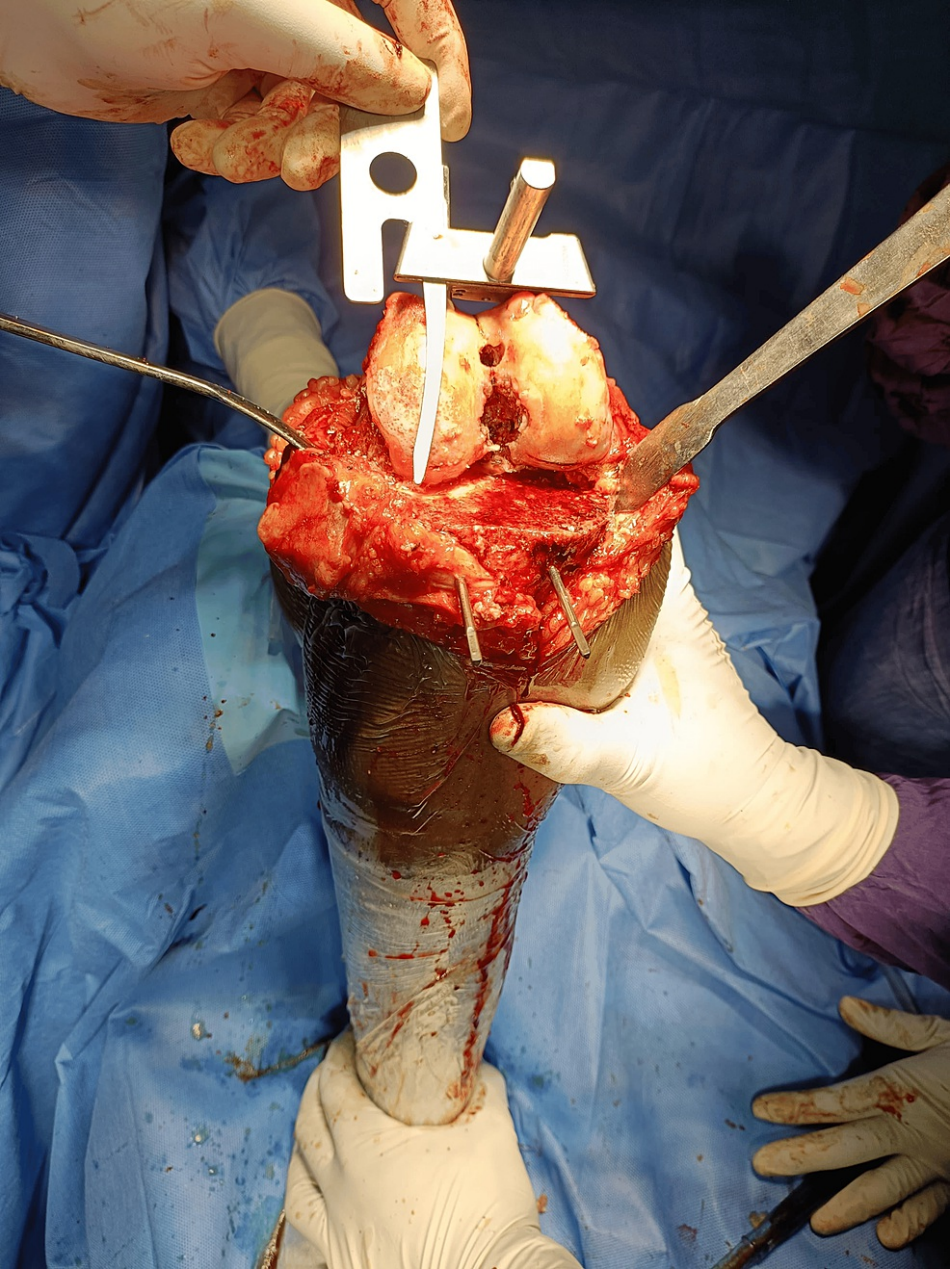
Measuring cuts

**Figure 4:**
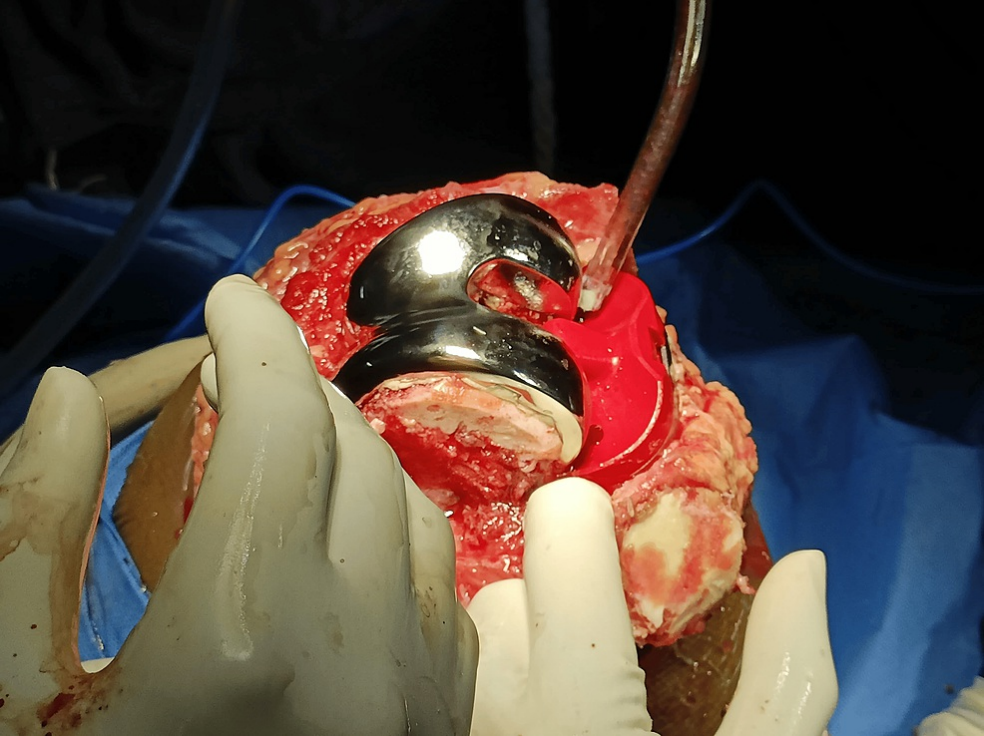
Cemented posterior stabilized total knee arthroplasty prostheses

**Figure 5:**
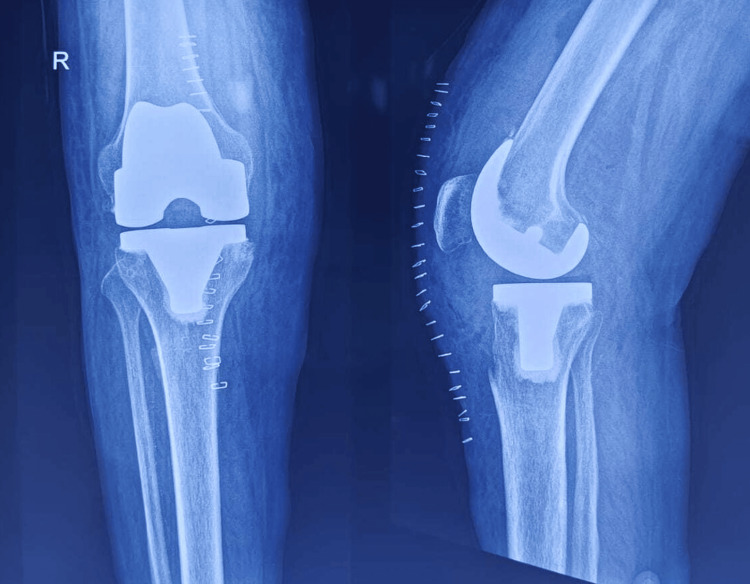
Radiograph after total knee arthroplasty with posterior stabilised prostheses

Preoperative templating was done based on plain radiographs and CT scanograms. Angles were calculated using the Angulus Android application, as seen in Figure 1. A medial parapatellar approach (Figure 2) with an inside-out soft tissue release of the posterolateral capsule and complex with pie crusting of the iliotibial band was employed, along with resection of the proximal part of the tibia and distal part of the femur to provide a balanced rectangular space [2-4]. After using the gap balancing technique, bony cuts were taken (Figure 3). Cemented, posterior stabilized implants (DePuy Synthes SIGMA® Primary Knee System, Raynham, MA) were used in all knees (Figure 4). Clinical and radiographic evaluation (Figure 5) was performed at one, three, six, and 12 months postoperatively and functional outcomes were measured based on the range of motion, KSS, and degree of deformity.

Statistical analysis

All statistical analyses were performed using SPSS version 22.0 software (IBM Corp., Armonk, NY). All variables were assessed for normality of distribution and coded before data analysis. Comparisons of continuous variables between two groups were made using the paired t-test for normally distributed data or the Wilcoxon signed rank sum test for non-normally distributed data with p < 0.05 being considered significant.

Presentation of cases

One senior surgeon performed all of the procedures in the same hospital utilizing epidural or general anaesthesia with a tourniquet. Out of six cases, five were males and one was female, all having valgus knee deformity secondary to osteoarthritis. All cases had grade 2 valgus deformity according to the Ranawat classification and had a limited range of motion preoperatively. All the patients had a valgus thrust gait and medial collateral ligament laxity and underwent TKA using non-constrained implants and soft tissue releases. Postoperative assessment and one-year follow-up were done clinically through the Knee Society Scoring system and radiologically through deformity angle measurement via the Angulus Android app.

Case 1

**Figure 6:**
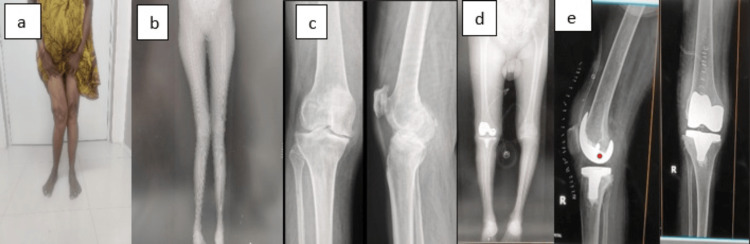
Case 1 (a) Preoperative clinical photograph. (b) Preoperative scanogram. (c) Preoperative radiograph. (d) Postoperative scanogram. (e) Postoperative radiograph.

A 55-year-old female presented with complaints of pain in both knees with the right more than the left and difficulty in walking for one year. She was diagnosed with bilateral knee osteoarthritis with a 13.4-degree valgus deformity on the right side (Figures 6A-6C). Clinically the valgus stress test revealed medial side laxity. Preoperative range of motion was 65 degrees and KSS was found to be 48.

Following preoperative evaluation, the patient was posted for right total knee replacement surgery via medial parapatellar approach along with soft tissue release of the posterolateral capsule and complex. The cemented posterior stabilized implant was kept as seen in Figure 6E. Postoperatively, the angle of deformity was found to be 5.9 degrees (Figure 6D). The patient was followed up at regular intervals postoperatively and the KSS was found to be 96. The range of motion was found to be improved to 111 degrees.

Case 2

**Figure 7:**
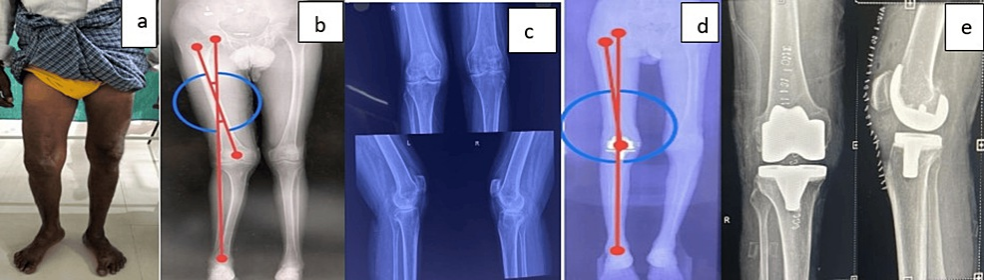
Case 2 (a) Preoperative clinical photograph. (b) Preoperative scanogram. (c) Preoperative radiograph. (d) Postoperative scanogram. (e) Postoperative radiograph.

A 60-year-old male presented with complaints of pain in the right knee and difficulty in walking for eight months. He was diagnosed with right knee osteoarthritis with a grade 2 valgus deformity of 12.9 degrees based on radiographic evaluation (Figures 7A-7C). Clinically, the valgus stress test was positive. The preoperative range of motion was found to be 52 degrees. Preoperative KSS was 45.

Right total knee replacement surgery was done through a medial parapatellar approach along with inside-out soft tissue release of the posterolateral capsule and complex with pie crusting of the iliotibial band. Non-constrained cemented posterior stabilized implant was used as seen in Figure 7E. The angle of deformity was found to be 5.9 degrees postoperatively (Figure 7D). The patient was followed up at regular intervals postoperatively and the KSS was found to be 94. The range of motion was found to be improved to 107 degrees.

Case 3

**Figure 8:**
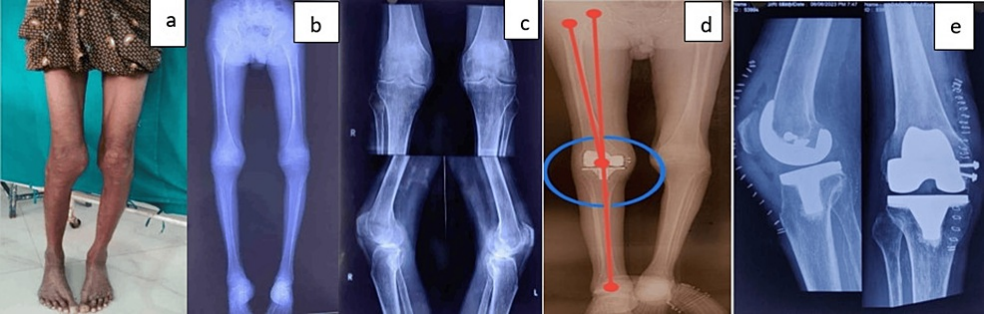
Case 3 (a) Preoperative clinical photograph. (b) Preoperative scanogram. (c) Preoperative radiograph. (d) Postoperative scanogram. (e) Postoperative radiograph.

A 56-year-old male presented with complaints of right knee pain and difficulty in walking for over nine months. He was diagnosed with right knee osteoarthritis with grade 2 valgus deformity and an angle of deformity of 10 degrees based on radiographic evaluation (Figures 8A-8C). Valgus stress test revealed medial side laxity and the preoperative range of motion was limited to 72 degrees. Preoperative KSS was found to be 43.

Following preoperative evaluation, the patient was posted for right total knee replacement surgery via medial parapatellar approach along with soft tissue release. Intraoperative medial condyle femur fracture was stabilized via two cannulated cancellous screws (Figure 8E). The cemented posterior stabilized implant was kept as seen in Figure 8E. Postoperatively, the angle of deformity was found to be 5.2 degrees (Figure 8D). The patient was followed up at regular intervals postoperatively and the KSS was found to be 95. The range of motion was also found to be improved to 109 degrees.

Case 4

**Figure 9:**
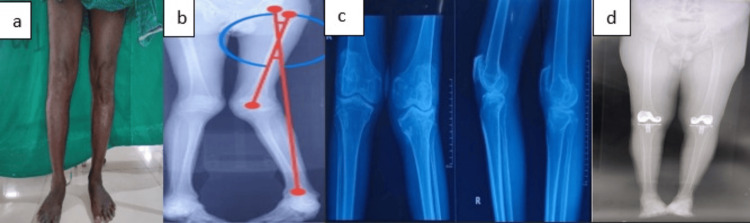
Case 4 (a) Preoperative clinical photograph. (b) Preoperative scanogram. (c) Preoperative radiograph. (d) Postoperative scanogram.

A 56-year-old male presented with bilateral knee pain and difficulty in walking for nine months. Based on the clinical findings of medial compartment laxity and the radiological findings of 10 degrees valgus deformity, he was diagnosed as having grade 2 genu valgus left secondary to osteoarthritis knee as seen in Figures 9A-9C. Preoperative KSS was found to be 44 and preoperative range of motion was 58 degrees

Following preoperative evaluation, the patient was posted for bilateral total knee replacement surgery via medial parapatellar approach along with lateral soft tissue release for the left valgus side, which was included for analysis. The cemented posterior stabilized implant was used. Postoperatively, the tibiofemoral angle was found to be 5.2 degrees (Figure 9D). The patient was followed up at regular intervals postoperatively and the KSS was found to be 95. The range of motion was also found to be improved to 106 degrees.

Case 5

**Figure 10:**
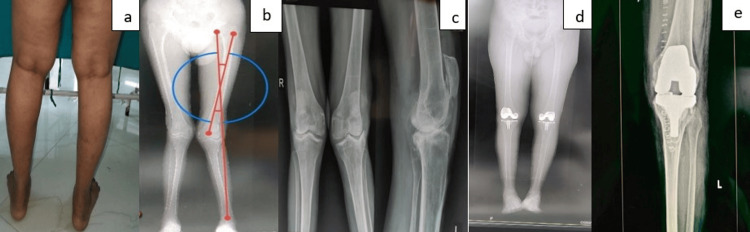
Case 5 (a) Preoperative clinical photograph. (b) Preoperative radiograph. (c) Preoperative scanogram. (d) Postoperative scanogram. (e) Postoperative radiograph.

A 64-year-old male presented with complaints of pain in both knees and difficulty in walking for 18 months. He was diagnosed with bilateral knee osteoarthritis with grade 2 valgus deformity of the left side and an angle of deformity of 12.9 degrees based on radiographic evaluation (Figures 10A-10C), and clinically, valgus stress test revealed medial side laxity with a range of motion limited to 45 degrees. Preoperative KSS was found to be 46.

Following preoperative evaluation, the patient was posted for bilateral total knee replacement surgery via medial parapatellar approach along with lateral soft tissue release for the left valgus side. The cemented posterior stabilized implant was used, as shown in Figure 10E. The left valgus knee alone was included for purposes of analysis. Postoperatively, the angle of the deformity was found to be 6.2 degrees (Figure 10D). The patient was followed up at regular intervals postoperatively and the KSS was found to be 96. The range of motion was also found to be improved to 112 degrees.

Case 6

**Figure 11:**
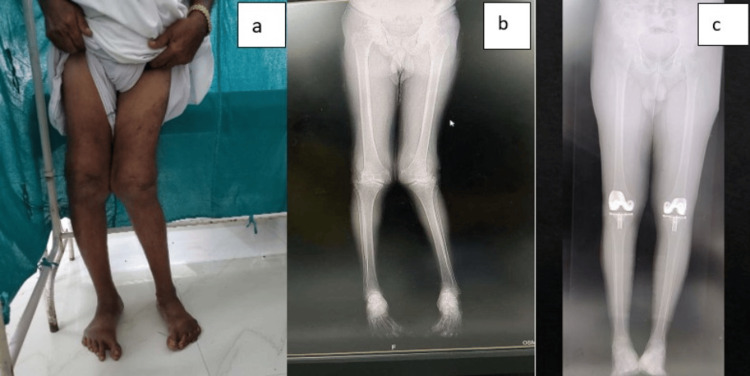
Case 6 (a) Preoperative clinical photograph. (b) Preoperative scanogram. (c) Postoperative scanogram.

A 70-year-old male presented with complaints of pain in both knees and difficulty in walking for 11 months. He was diagnosed with bilateral knee osteoarthritis with grade 2 valgus deformity of the left side and an angle of deformity of 13.2 degrees (Figures 11A, 11B). Preoperative range of motion was 68 degrees and KSS was found to be 48.

Bilateral total knee replacement surgery was done through a medial parapatellar approach along with inside-out soft tissue release of the posterolateral complex with pie crusting of the iliotibial band on the left valgus side. The left valgus knee alone was considered for analysis. Non-constrained cemented posterior stabilized implant was used. The angle of deformity was found to be 5.1 degrees postoperatively (Figure 11C). The patient was followed up at regular intervals postoperatively and the KSS was found to be 94. The range of motion was also found to be improved to 108 degrees.

## Results

This study included six patients who underwent TKA. The mean age and standard deviation were 61.16 ± 5.52 (55-70) years and out of six cases, five were males and one was female.

The mean and SD of variables were found to be age = 61.16 ± 5.52 years, angle of deformity = 12.55 ± 1.77 degrees, angle after surgery = 5.75 ± 0.14 degrees, KSS before and after surgery = 47 ± 1.67 and 95.50 ± 0.89, respectively, and the range of motion (ROM) was 109.04 ± 0.16 (Table 1).

**Table 1:** Descriptive statistics of the case series KSS: Knee Society Score.

Variables	N	Mean	Std. deviation
Age	6	61.16	5.52
Angle of deformity (before surgery)	6	12.55	1.77
Angle of deformity (after surgery)	6	5.75	0.14
KSS (before surgery)	6	47.00	1.67
KSS (after surgery)	6	95.50	0.89
Range of motion (before surgery)	6	60.00	10.25
Range of motion (after surgery)	6	109.04	0.16

Table 2 shows the paired T-test that was performed on the tibiofemoral angle before and after surgery with a mean difference of 6.80 degrees and a t-value of 9.130 at a significance level of p-value < 0.001 indicating a statistically significant difference between the angle of deformity before and after surgery. The t-value of 9.130 is much higher than the critical value, suggesting strong evidence against the null hypothesis, which implies that the surgery has effectively reduced the valgus deformity (mean and SD of the angle of the deformity was 12.55 ± 1.77 degrees and 5.75±0.14 degrees before and after TKA, respectively).

**Table 2:** Evaluation of significance mean difference before and after TKA surgery using paired t-test statistics T: paired t-test value; df: degrees of freedom; TKA: total knee arthroplasty; KSS: Knee Society Score.

Paired samples test										
Variables		Paired differences					T	df	Significance	
		Mean	Standard deviation	Standard error mean	95% confidence interval of the difference				One-sided p-value	Two-sided p-value
					Lower	Upper				
Pair 1	Angle of deformity pre and post-TKA	6.80	1.82	0.74	4.88	8.71	9.130	5	<0.001	<0.001
Pair 2	KSS clinical score pre and post-TKA	-48.50	-0.8	-0.67	-45.224	-41.77	-67.97	5	<0.001	<0.001

Similarly, analysis of the KSS before and after surgery showed a mean difference of -48.50 and a t-value of 67.97 at a significance level of p-value < 0.001, also indicating a statistically significant difference. The high t-value suggests a significant improvement in KSS after surgery, indicating that the surgery substantially enhanced knee function and overall patient satisfaction (since knee range of movement and patient satisfaction are components of the KSS). The paired t-tests showed a highly significant improvement after surgery in terms of the angle of deformity and knee function as measured by the KSS standard scale. The average range of motion at the knee joint after surgery was 109 degrees (Table 3).

**Table 3:** Evaluation of significance between preoperative and postoperative range of motion ROM: range of motion; T: paired t-test value.

Paired samples test							
Variables		Mean	N	Std. deviation	Std. error mean	T-value	P-value
Pair 1	Preoperative ROM	60.0000	6	10.25671	4.18728	-11.657	<0.001
	Postoperative ROM	109.0467	6	0.11501	0.04695		

The paired samples statistics illustrate a significant difference between preoperative range of motion (mean = 60.0000, SD = 10.25671) and postoperative range of motion (mean = 109.0467, SD = 0.11501) scores. The t-value of -11.657 with a p-value of less than 0.001 indicates a statistically significant change between the two time points. This suggests that there is a substantial difference in the measurements taken before and after the intervention (preoperative range of motion and postoperative range of motion, respectively), highlighting the effectiveness or impact of the intervention on the observed outcome.

## Discussion

One of the most often performed and most dependable surgical operations in the USA is TKA due to its success rate [11]. It is projected that 3.48 million procedures will be performed yearly in 2030 (up 601% from 2005 to 2030) [7].

A successful TKA operation should result in a neutral alignment of the knee in the coronal plane, which means the bone cut should be orthogonal to the mechanical axis and the normal knee typically has 6° of valgus angle [12]. In cases of the valgus knee, the presence of both femoral and tibial pathology should be addressed following stabilization. According to Lee et al., under-correction during TKA may result in a residual valgus angle greater than 6°, which can lead to patellar mal tracking and a lower KSS [13]. In our study, we have achieved a mean postoperative tibiofemoral angle of 5.7 degrees valgus, where 4-6 degrees valgus is considered normal for healthy individuals [13].

The present study comprises six patients with a mean age of 61 years and a mean angle of deformity of 12.55 ± 1.77 degrees before the TKA and 5.75 ± 1.14 after the surgery, with a mean improvement difference of 6.80 degrees, with significance of p < 0.001. These results are similar to the statements of Greenberg et al., where the valgus deformity was corrected from 16.2 ± 5.6 to 5.4 ±2.8 (p < 0.001) with TKA [14].

Ranawat et al. in their seminal study detailing the treatment and follow-up of patients with valgus deformities of the knee mentioned that a trial with less constrained prostheses was advisable for those with grade 2 deformities and below [3].

Another study conducted by Ren et al. utilized the least constrained prostheses possible in 62 severe type 2 valgus knee deformity patients who underwent TKA, and they showed good results with improvements in average valgus degree was 7.3° and range of motion averaged at 110.6°; in our study, the mean range of motion was found to be 109.04° after TKA [15].

The fact that Utomo et al. used a non-constrained TKA implant approach to treat osteoarthritis with grade 2 valgus and were able to create a stable, full correction of the patient's valgus deformity within three months after surgery further supports this method [16].

All patients' KSS considerably improved as well with a mean average of 47 ± 1.67 and 90 ± 1.64 pre- and post-TKA, respectively, with a mean difference of 43.5 (p < 0.001). A proven tool for evaluating patient function, expectations, symptoms, and satisfaction following TKA is the KSS system [9,10]. When doing a TKA, there are a few different techniques, including the lateral and medial parapatellar approaches. Many surgeons prefer to perform a lateral parapatellar approach for fixed valgus deformities, but this approach is technically demanding and often requires an additional tibial tubercle osteotomy to be performed due to the difficulty faced with medial eversion of the patella [6].

Another study conducted in 2004 by Elkus et al., demonstrated that TKA using the medial parapatellar approach improved alignment in support of this study, which improved from 12.55 degrees of valgus preoperatively to 5.7 degrees postoperatively and KSS also improved, going from 47 points preoperatively to 95 points postoperatively [2]. According to Lombardi et al., the medial parapatellar technique yields a satisfactory long-term result, which also supports this study [6].

We sought to bolster the findings of others that valgus deformities can be satisfactorily addressed during a TKA via the easier medial parapatellar approach, as we demonstrated in our six cases. This study's small number of subjects is a limitation that could be addressed in future research to strengthen the evidence further in this regard. Another limitation of the study is the short period of follow-up as long-term complications and outcomes could not be assessed in this time frame, evaluation with more assessment and a longer evaluation period would be needed to extrapolate these findings to the general population. Further limitations include the fact that we only included grade 2 valgus deformities in our study, the exclusion of valgus deformities secondary to rheumatoid arthritis, and the fact that we used the old KSS system rather than the newer KSS system released in 2011, which includes subjective components such as satisfaction and fulfilment of patient's expectations [17].

## Conclusions

A valgus deformity secondary to osteoarthritis in the knee presents a unique challenge for total knee replacement. We found that TKA with the medial parapatellar approach in the valgus knee provides good clinical and radiological results with satisfactory deformity correction and improved knee alignment with good ROM in our series. Positive clinical and radiological results in our patients serve as evidence of this. After one year of TKA, the KSS showed a drastic improvement. A larger sample size and a longer evaluation period are needed to further confirm and determine the effectiveness of this strategy.
